# Combined endothelial and epithelial barrier disruption of the colon may be a contributing factor to the Ebola sepsis-like syndrome

**DOI:** 10.1186/s13037-014-0048-z

**Published:** 2015-01-23

**Authors:** Lawrence A Lynn

**Affiliations:** The Sleep and Breathing Research Institute, 1275 Olentangy River Road, Suite 10, Columbus, Ohio 43212 USA

**Keywords:** Ebola, Sepsis, Translocation, Bacteremia, Endotoxin, Decontamination, Antibiotics, Intestine, Cytokines, Colon

## Abstract

After an initial febrile viral syndrome, infection with Ebola virus often induces an explosive late “Ebola sepsis-like syndrome” which appears very similar to some phenotypes of bacterial sepsis and is commonly fatal. It is possible that direct and diffuse viral infection of both the endothelium and epithelium of the colon may cause sufficient disruption of both the endothelial and epithelial barriers to induce exposure or leakage of endotoxin and bacterial antigens to, or into, the vascular system precipitating or exacerbating the Ebola sepsis-like syndrome. If colonic barrier disruption or vascular exposure of bacterial antigens from the colon is found to comprise an important mechanism of the Ebola sepsis-like syndrome, protocolized timed decontamination of the bowel with or without timed prophylactic antibiotics might warrant investigation.

**Electronic supplementary material:**

The online version of this article (doi:10.1186/s13037-014-0048-z) contains supplementary material, which is available to authorized users.

## Letter to the Editor

I studied the relational timed data comprised of reanimated relational motion images of clinical data derived from Ebola virus (EBOV) infection in two published naturally infected human cases [1,2] and nine intentionally infected rhesus macaques [3]. To achieve reanimation, the clinical data from these published cases were entered into a new type of medical software which converts the entire lab, vitals and other clinical data of a patient into a time series matrix. The software then detects primary and relational perturbations of along the different time series which comprise the matrix. When multiple relational perturbations occur together (for example in a condition such as sepsis) this comprises a distortion of the time matrix and the software detects this distortion and converts it into a motion image, such as a color radar weather video, which changes over time in response to the evolution of the clinical condition. This provides a time lapsable view of the relational patterns which comprise complex clinical conditions (Additional file 1).

**Figure 1:**
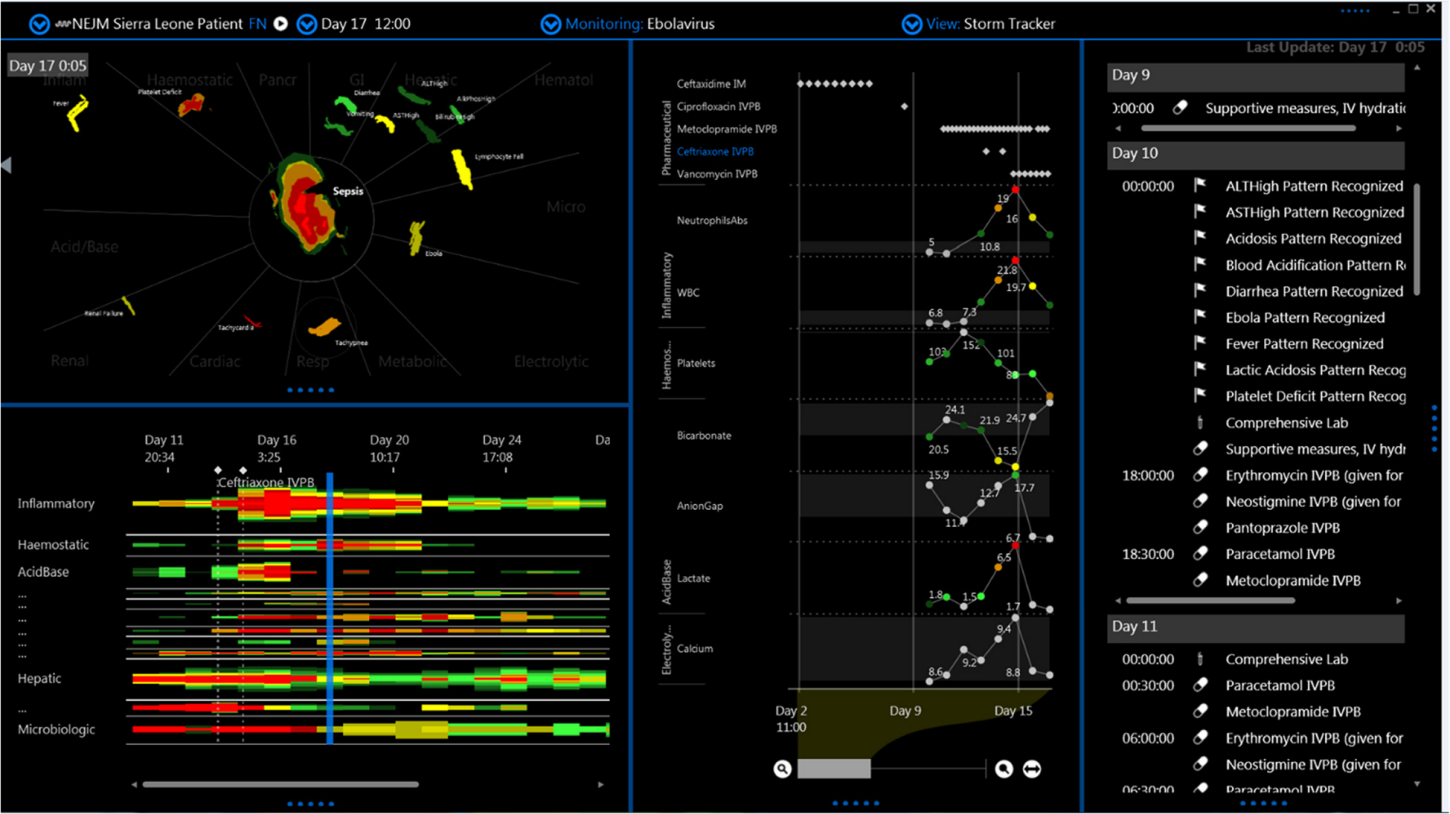
**Screen capture of motion image of Zaire Ebola virus infection.** This is a screen capture of a time-lapsed video which was reanimated from data published in reference 1. The left lower panel shows a time map of perturbations of laboratory and vitals datasets divided into systems (the inflammatory system is on the top). The left upper panel is a weather map visualization of the time matrix distortion showing the image at the point of the blue line on the time map. The right panel shows the time series of Ebola viral load and antibody response. An apparent Ebola sepsis like syndrome (ESLS) emerges on day 14 with a precipitous onset (see rapid distortion of the inflammation system on the time map on day 14). While the pattern looks similar to ESLS, as noted by the authors it is likely that this distortion was primarily caused by enteric bacterial sepsis.

**Figure 2:**
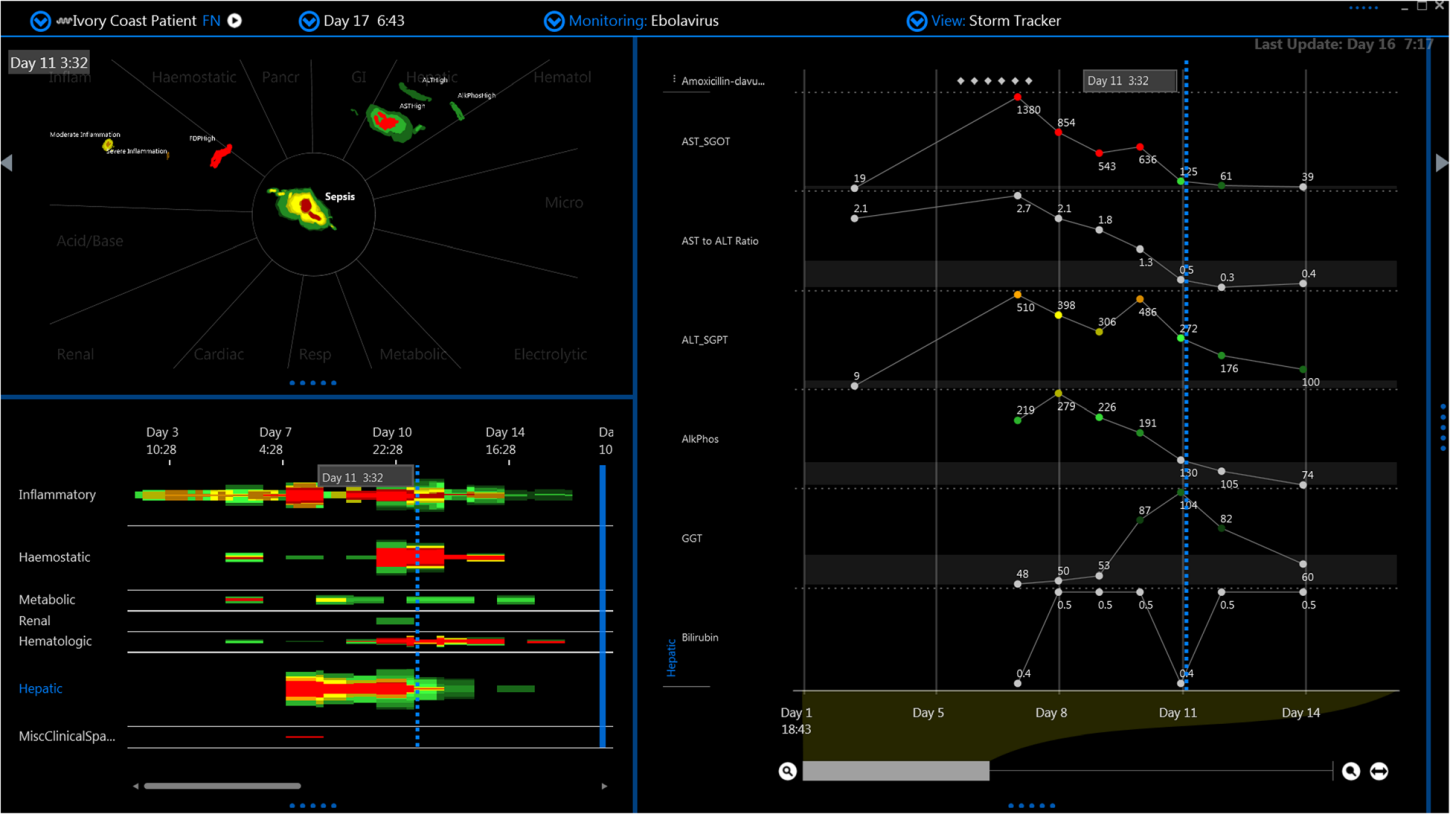
**Screen capture of Ebola virus subtype Côte d'Ivoire infection from day 1 to day 16.** This is a screen capture of a time-lapsed video which was reanimated from data published in reference in reference 2. The ESLS occurs on day 7. No polymerase chain reaction (PCR) data are available in this case.

On observation of the motion images of the published datasets derived from Ebola infection, one striking feature of EBOV infection which was observed in most cases was the precipitous and late development of a motion image similar to some phenotypes of bacterial sepsis. This was not surprising as EBOV infection is known to produce a mixed anti-inflammatory response syndrome (MARS), and a late Ebola sepsis-like syndrome (ESLS) similar to some common phenotypes of bacterial sepsis [4]. However, as evident in Figure 1 and Figure 2, the ESLS may occur after a prolonged period of a milder febrile viral syndrome and may be absent for many days despite a high Ebola virus load. Perhaps the most striking feature of the case presented in Figure 1 is the failure of the patient to initially exhibit ESLS despite high and prolonged viral load. For example, on the initial day 10 a sepsis-like distortion of the matrix is not present despite severe plasma vial load of 7.98 log10 (copies/ml). In fact, the onset of an apparent ESLS occurs precipitously 4 days later and after viral load began to fall. In this case ESLS appears to be more associated with the positive culture for an enteric organism taken on day 12 then with the Ebola viral load suggesting that this sepsis pattern was not ESLS but rather actual enteric bacterial sepsis.

This raises an important point. What is the difference between the dynamic relational clinical pattern, the matrix distortion phenotype, of ESLS and the common matrix distortion phenotypes of enteric bacterial sepsis? Might ESLS actually be a response to both viral and enteric antigens? Is an additional bacterial source for cytokine activation in present in many or all cases of ESLS?

**Figure 3:**
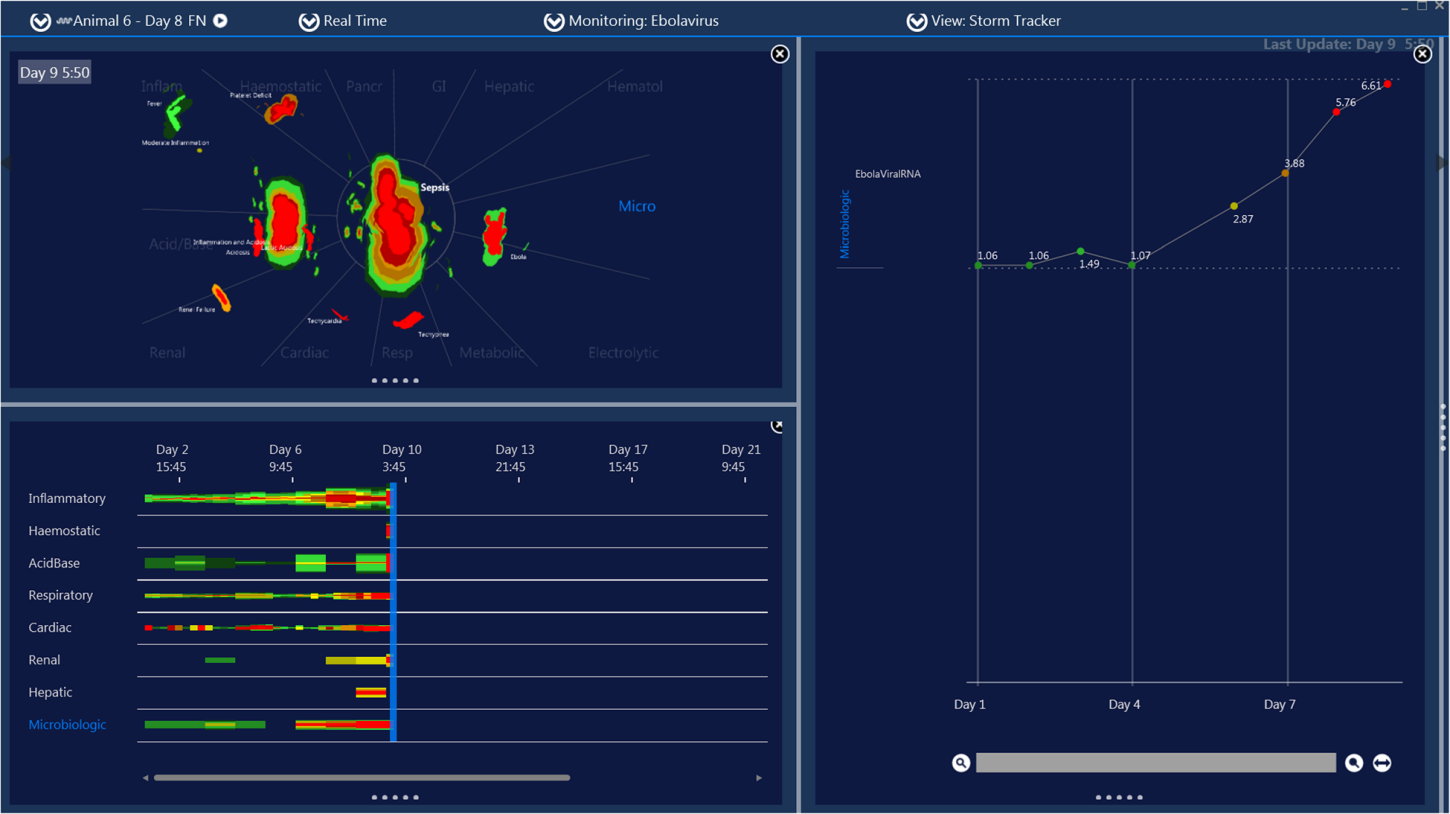
**Screen capture of motion image of Zaire Ebola virus infection in a rhesus macaque.** This is Animal 6 in reference 2. This animal develops severe and rapidly fatal ESLS which coincides with peak viral RNA levels on day 9.

**Figure 4:**
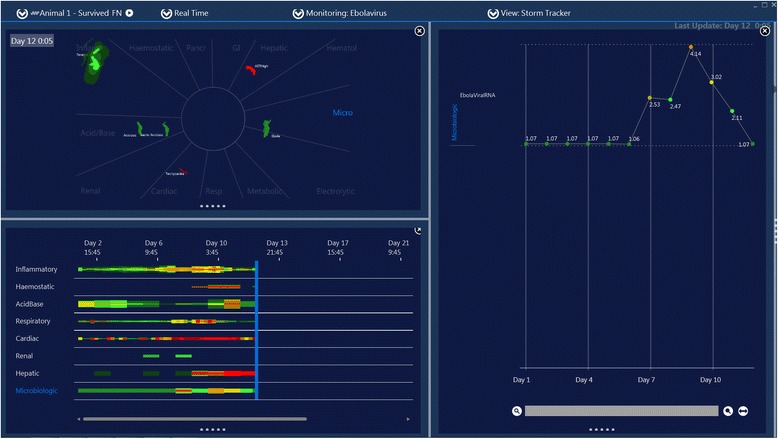
**Screen capture of motion image of Zaire Ebola virus infection in a rhesus macaque.** This is Animal 1 in reference 2. Note this animal never develops the ESLS despite high viral RNA levels.

**Figure 5:**
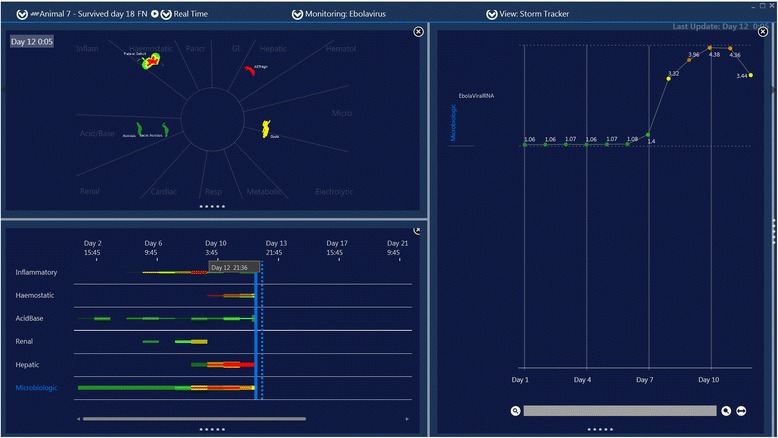
**Screen capture of motion image of Zaire Ebola virus infection in a rhesus macaque.** This is Animal 7 in reference 2. Note this animal never develops the ESLS despite high viral RNA levels.

In some examples such as the case in Figure 3 the ESLS appears to develop in direct relation to the rise and peak of viral RNA. However in others (Figure 4 and Figure 5) ESLS never develops despite high viral RNA. Other macaques in the study [3] demonstrated similar disassociation between the development of ESLS and viral RNA.

There are many explanations for these relational discrepancies between vial load and ESLS seen in some patients and animals. However it is not unreasonable to entertain that at least a portion of ESLS in most, or perhaps virtually all, patients is of bacterial rather than viral origin. Given these striking patterns, it is reasonable to speculate that the at least some EBOV genotypes may have evolved to selectively infect and damage colon epithelium and endothelium allowing escape of endotoxin and bacterial antigens associated with bacteria living from the lumen of the colon into the plasma to thereby prolong the period and magnitude of viral infectivity in the living host. This may also serve the interest of the viral genome by increasing the probability of death of the host before viral clearance, thereby also increasing the probability of infection of other humans who touch the dying patient or contact the corpse. It is also possible that in more severe cases, enteric organism may cross this disrupted barrier of the colon producing a bacterial sepsis phenotype which overlaps with the mixed anti-inflammatory response syndrome already induced by the Ebola virus.

**Figure 6:**
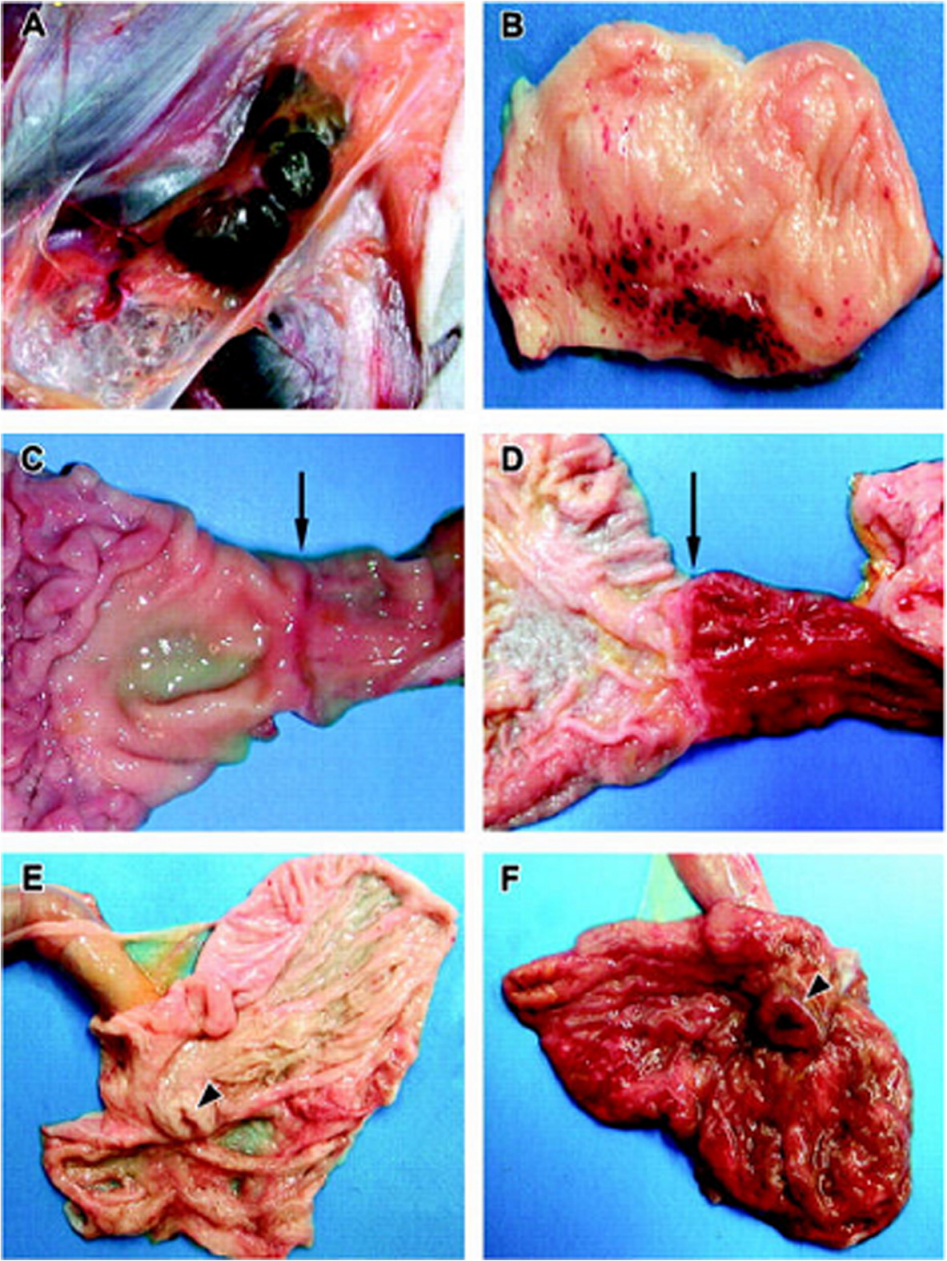
**Illustration of progression of inflammation/injury of the duodenum and colon induced by Ebola Virus.** Figure from reference 5, used with permission. Representative gross necropsy lesions from cynomolgus monkeys experimentally infected with EBOV-Zaire. **A**: Mild enlargement and marked congestion/hemorrhage of inguinal lymph nodes at day 4. **B**: Multifocal to coalescing hemorrhages of mucosa of urinary bladder at day 5. **C** and **D**: Progression of marked congestion of the duodenum occurring between day 3 **(C)** and day 5 **(D)**. Arrows indicate the gastroduodenal junction demarcating the stomach to the left and the duodenum to the right. The duodenum is markedly congested at day 5 **(D)**, **E** and **F**: Progression of congestion of cecum occurring between day 3 **(E)** and day 5 **(F)**. The cecum is opened up and the ileum extend outward from the cecum. Arrowheads indicate the ileocecal junction. Note the congested and thickened appearance of the cecum at day 5 (F).

Geisbert et al. [5] (Figure 6) show striking evidence of diffuse injury/inflammation of the colon and duodenum. The timed progression of this process from day 3 to day 5 is dramatic. When considered visualizing liquid stool adjacent the nascent areas the diffuse injury of the cecum (Arrow of Figure 6) the potential for transfer of bacterial antigens is evident as the injury induced by Ebola virus is not limited to epithelium. It seems possible that direct and diffuse viral infection of both the endothelium and epithelium of the colon may, over time, cause sufficient injury and associated disruption of the tight junctions of both the endothelial and epithelial barrier to allow movement of large molecules such as endotoxin and bacterial antigens into close proximity with, or into, the vascular system. This may precipitate an explosive cytokine cascade and an ESLS which may appear virtually identical to some phenotypes of enteric bacterial sepsis. This could be allowing EBOV to generate its fatal ELSL using enteric bacterial antigenic surrogates despite an otherwise timely onset of plasma viral clearance associated with the emergence of EBOV IgG and IgM antibodies.

This raises the possibility that a properly timed protocol of bowel decontamination (perhaps in combination with oral antibiotics effective for enteric organism) might be effective in mitigating the development or severity of ESLS. This might comprise something as simple as a protocol for timed mechanical bowel preparation/decontamination. For example, full decontamination and/or prophylactic oral antibiotics might begin a select number of days after the onset of fever, or upon the onset of diarrhea.

At this point, both the cause of the ESLS and the efficacy and toxicity of timed bowel preparation/decontamination remains speculative and such treatment would require study in a clinical trial. Risks associated with bowel decontamination in this setting include the potential increase of diarrhea, which has already been documented to approach 8 liters a single day [1], the potential for the development of super infection with Clostridium difficile or other organisms, and toxic diffusion of bowel decontaminating medication across the disrupted endothelial/epithelial intestinal barrier.

## Conclusion

It is possible that some strains of Ebola virus have evolved a mechanism to amplify the virulence of their genome by specifically infecting the endothelial and epithelial barriers of the colon and thereby inducing movement of large molecules such as bacterial endotoxin from the lumen of the colon into the host vasculature. Investigation is warranted to identify if this is an important mechanism of the late sepsis- like syndrome commonly induced by Ebola virus infection. If this mechanism is identified, properly timed mechanical bowel preparation/decontamination with or without properly timed prophylactic oral antibiotics, may warrant investigation as a means to mitigate the Ebola sepsis-like syndrome.

## Additional file

Additional file 1:
**This file shows a time-lapsed video of distortions of the time-matrix of the patient in reference 1.** An audio explanation of the video is included.

## Abbreviations

EBOV: Ebola virus
ESLS: Ebola sepsis-like syndrome
PCR: Polymerase chain reaction

## References

[1] Kreuels B, Wichmann D, Emmerich P, Schmidt-Chanasit J, de Heer G, Kluge S, Sow A, Renné T, Günther S, Lohse AW, Addo MM, Schmiedel S: A Case of Severe Ebola Virus Infection Complicated by Gram-Negative Septicemia. N Engl J Med. 2014; 371(25):2394-401. 10.1056/NEJMoa1411677 25337633

[2] Formenty P, Hatz C, Le Guenno B, Stoll A, Rogenmoser P, Widmer A (1999). Human infection due to Ebola virus, subtype Côte d'Ivoire: clinical and biologic presentation. J Infect Dis.

[3] Kortepeter MG, Lawler JV, Honko A, Bray M, Johnson JC, Purcell BK, Olinger GG, Rivard R, Hepburn MJ, Hensley LE (2011). Real-time monitoring of cardiovascular function in rhesus macaques infected with Zaire ebolavirus. J Infect Dis.

[4] Ebihara H, Rockx B, Marzi A, Feldmann F, Haddock E, Brining D, LaCasse RA, Gardner D, Feldmann H (2011). Host response dynamics following lethal infection of rhesus macaques with Zaire ebolavirus. J Infect Dis.

[5] Geisbert TW, Hensley LE, Larsen T, Young HA, Reed DS, Geisbert JB, Scott DP, Kagan E, Jahrling PB, Davis KJ (2003). Pathogenesis of Ebola hemorrhagic fever in cynomolgus macaques: evidence that dendritic cells are early and sustained targets of infection. Am J Pathol.

